# Genetic variations of body weight and GCRV resistance in a random mating population of grass carp

**DOI:** 10.18632/oncotarget.5945

**Published:** 2015-10-01

**Authors:** Rong Huang, Jiaxian Sun, Qing Luo, Libo He, Lanjie Liao, Yongming Li, Fuhua Guo, Zuoyan Zhu, Yaping Wang

**Affiliations:** ^1^ State Key Laboratory of Freshwater Ecology and Biotechnology, Institute of Hydrobiology, Chinese Academy of Sciences, Wuhan, China; ^2^ Tongwei Company, Limited, Chengdu, China

**Keywords:** Ctenopharyngodon idellus, full-sib families, paternity test, breeding, Pathology Section

## Abstract

The grass carp (*Ctenopharyngodon idellus*) is an important species in freshwater aquaculture both in China and on a global scale. Variety degeneration and frequent diseases have limited the further development of grass carp aquaculture. Thus, new and improved varieties are required. Here, we identified and assessed the body weight and disease resistance in a random mating population of 19 ♀ × 22 ♂ grass carp, which were derived from different water systems. In both the growth experimental group of 10,245 fish and grass carp reovirus (GCRV)-infected group with 10,000 fish, 78 full-sib families were statistically analyzed for body weight and GCRV resistance. The findings showed that body weight traits had low heritability (0.11 ± 0.04, 0.10 ± 0.03 and 0.12 ± 0.05), GCRV resistance traits had high heritability (0.63 ± 0.11); body weight was higher in 3 families, whereas GCRV resistance was significantly greater in 11 families. Our results confirmed that the natural germplasm resources of wild grass carp were genetically diverse. Breeding of GCRV resistant varieties of grass carp have better genetic basis. This study provides the basis for constructing basal populations for grass carp selective breeding, quantitative trait loci (QTL) and genome-wide association studies (GWAS) analysis.

## INTRODUCTION

The grass carp (*Ctenopharyngodon idellus*) is an important freshwater fish with the highest output in fish farming worldwide, and its production accounted for 15.6% of global freshwater aquaculture production in 2011 [1]. However, growth degradation and frequent diseases due to limited genetic resources have restricted its use in aquaculture [2, 3]. Grass carp reovirus (GCRV) is an important fish pathogen involved in hemorrhagic disease, mainly infecting young fish fingerlings and yearlings of grass carp and *Mylopharyngodon piceus* [4]. This virus is widespread in south China and causes severe economic devastating losses to national aquaculture industries [5]. Since the 1960s, various intra- and interspecific hybridization for breeding studies have been carried out and progress has been made in the breeding of growth performance and disease resistance. As an example, specimens selected from a population in Heilongjiang River in China increased the growth rate by 11.5% [6], whereas disease-resistant grass carp lines were harvested from hybridized generations between *Cyprinus carpio* and grass carp. This cross increased GCRV resistance by 54.69% [7], but has not been commercialized yet due to the genetic instability of the hybrid fish. Due to the long sexual maturation time (4-5 years) of grass carp, the traditional breeding cycle is long and inefficient; this is a common problem encountered in farmed fish breeding. Thus, it is critical to improve the breeding efficiency to promote the cultivation of varieties of fish [8, 9].

With the development of molecular markers and quantitative trait loci (QTL) genotyping methods, the marker-assisted selection (MAS) method has been applied in fish breeding, and has improved breeding efficiency [10, 11]. For example, Japanese researchers were able to breed a new anti-lymphocystis disease (LD) variety of *Paralichthys olivaceus* using a resistance allele of *Poli9-8TUF*, which showed no incidence of LD at either farm [12], while Norwegian researchers successfully bred a new variety of *Salmo salar* based on anti-infectious pancreatic necrosis (IPN) QTL with a death rate of only 13% after infection with IPNV, while the mortality in the control group reached up to 51.7-98.5% [13]. By using next-generation sequencing (NGS) technology, recent studies on genome sequencing and the functional genome have been performed on a variety of model species and fish species of economic importance (e.g. *Ctenopharyngodon idellus, Danio rerio, Oryzias latipes, Platanista minor, Gadus macrocephalus*) [1, 14-17]. The purpose of these studies was to reveal the genetic basis of complicated economic traits at the gene level, in order to rationalize the breeding process [18, 19].

Constructing the base population is a prerequisite for traditional and molecular breeding technologies and is critical for effective breeding. However, the construction of a base population for breeding requires a broad survey of germplasm resources and genetic analysis of desirable traits in fish lines for species lacking basic conditions for breeding, such as grass carp. It is therefore critical to improve the working efficiency. Since the 90's of 20th century, paternity test technology has been widely applied in fish farming and breeding [20]. Sugaya et al. could identify the genetic relationship of seven families of *Penaeus japonicas* by using five microsatellite loci [21]. From 110 potential parent pairs, the parents of 94.3% of *Salmo salar* were correctly identified with four microsatellite markers [22]. The use of paternity test technology has been shown to substantially improve the breeding efficiency [23, 24].

In the present study, a population was constructed artificially by randomly mating wild parents from different river systems. Combining paternity test technology, the heritability of bodyweight and GCRV resistance traits of the artificial random mating population was assessed. And the body weight and GCRV resistance of large-scale grass carp families were identified and evaluated. This study aimed to analyze the genetic diversity of grass carp, thus providing the basis for grass carp selective breeding, QTL and genome-wide association studies (GWAS) analysis.

## RESULTS

### Genetic diversity of wild-type grass carp parents

Detection of polymorphism in 41 wild-type grass carp parents showed that 16 pairs of primers obtained high quality amplification bands in 42 pairs of designed primers. These 16 loci were all polymorphic. 186 alleles were detected from 16 polymorphic loci in the test group, the number of alleles per locus ranged between 7-15, the average number of 16 loci was 11.625. The average effective number of alleles (NE), the average observed heterozygosity (HO), the average expected heterozygosity (HE) and average polymorphism information content (PIC) of these 16 polymorphic loci were 6.8988, 0.857, 0.8527 and 0.8249 respectively, the PIC values of 16 loci were all higher than 0.5, exhibited characteristics of high polymorphism (see Table 4).

**Table 1:** Origins and codes of wild grass carp parents

Male parent	Code	Source	Female parent	Code	Source
M1	41031B2153	Zhujiang river	F1	46271B6C48	Zhujiang river
M2	4103712C6A	Zhujiang river	F2	462658447B	Xiangjiang river
M3	4626781366	Yangtse river	F3	4627081A7D	Zhujiang river
M4	4440773345	Yangtse river	F4	462716060F	Xiangjiang river
M5	462812737B	Zhujiang river	F5	444073272D	Xiangjiang river
M6	46263F4A1A	Zhujiang river	F6	462811664F	Xiangjiang river
M7	4627133E2C	Zhujiang river	F7	20000014577	Yangtse river
M8	462735103B	Yangtse river	F8	4440743A53	Zhujiang river
M9	46266E3D0A	Zhujiang river	F9	4445200C39	Xiangjiang river
M10	46281E5A46	Xiangjiang river	F10	4626590622	Xiangjiang river
M11	46272A753C	Xiangjiang river	F11	46263F3C30	Xiangjiang river
M12	4627177F7A	Zhujiang river	F12	46271E1705	Xiangjiang river
M13	4627336177	Zhujiang river	F13	46270B3368	Zhujiang river
M14	20000015247	Yangtse river	F14	4627316D4C	Zhujiang river
M15	46266A4C21	Zhujiang river	F15	20000017502	Yangtse river
M16	4445220B53	Xiangjiang river	F16	41032C0912	Xiangjiang river
M17	44451B2734	Yangtse river	F17	020000014953	Lao river
M18	462837264A	Xiangjiang river	F18	020000017073	Lao river
M19	41033D1071	Zhujiang river	F19	4627004566	Zhujiang river
M20	41035B771B	Xiangjiang river			
M21	4627385968	Xiangjiang river			
M22	4626501D4E	Zhujiang river			

**Table 2:** Primers used for 16 microsatellite loci amplification

Locus	Primer^+^	Primer^−^	Allelic range
G023	m^a^ATAATACAAAAGTGTGGGAAGC	GAACAAGGTCCGATGCTAAT	177-312
G5002	mTAGTCTTCCACAAATCCAGTT	CCTGCTACTGTTACAAGTTTTT	219-269
G5004	mGGTCAAACCTTTCGTCAAT	TTTTATGCAGGAACTCTTACTAC	227-277
G5006	mATTCTGCTTTACTTTATGACACG	AGGTTTTATTTTGCCATACATTT	106-136
G5010	mCATTTTACTGCTTGCCTCAC	CCCTTCCTTTCGCATAGA	354-409
G5011	mAAGCCACCAACCTCTACGA	TAACAGGGATGGGATGAAAT	445-515
G5012	mGATGACATGGGGGTGAGTAA	CAGAAAGGTAGTAAACAACGAAA	319-369
G5020	mCAACCCTGTTTCTGTCCTGT	GCAAGCAACTGTCAACCTG	312-362
G5023	mCCAAGACCCAGTGGAAAC	CTCTACTGGCCTCTAGTGTGA	126-181
G5024	mATTCCTTCCGAAATCAGTG	AGTGGTGATGCTATTGCCT	196-251
G5025	mTCTACAAAGACCTTCGCTGA	AGTGGTGATGCTATTGCCT	355-425
G5028	mTGAAATCTGCATCTCCCTT	CCCTTCAGCCGTGACTTAT	197-242
G5034	mATCAGACACACTATGACAATGG	TGGAGGTTAGCAGGAAATAC	151-231
G5035	mCAAAACCTTGCTGTATTTAGAC	AAGGGTTACATTGAAATAGCAG	200-260
G5036	mACTTTTTGTTAACCTAAACCTCT	ACACACCCTCACATACACTCTC	173-228
G5037	mATTACACATGCTTAATGGGA	TTTGACAGACCTGGAACTTT	151-216

a “m” in the primer represents the sequence of M13 (CACGACGTTGTAAAACGAC).

**Table 3:** Primer sequences used for multiplex PCR

PCR combination	Locus	Primer^+^	Primer^−^	Fluorescent modification
Multiplex PCR in Group1	G5004	GGTCAAACCTTTCGTCAAT	TTTTATGCAGGAACTCTTACTAC	5′ROX
	G5006	ATTCTGCTTTACTTTATGACACG	AGGTTTTATTTTGCCATACATTT	5′ROX
	G5010	CATTTTACTGCTTGCCTCAC	CCCTTCCTTTCGCATAGA	5′ROX
Multiplex PCR in Group 2	G5011	AAGCCACCAACCTCTACGA	TAACAGGGATGGGATGAAAT	5′TAMRA
	G5023	CCAAGACCCAGTGGAAAC	CTCTACTGGCCTCTAGTGTGA	5′FAM
	G5024	ATTCCTTCCGAAATCAGTG	AGAGGGAGAAAGATAAGACCA	5′HEX
Multiplex PCR in Group 3	G5020	CAACCCTGTTTCTGTCCTGT	GCAAGCAACTGTCAACCTG	5′FAM
	G5036	ACTTTTTGTTAACCTAAACCTCT'	ACACACCCTCACATACACTCTC	5′ROX

**Table 4:** Genetic parameters of 16 microsatellite loci in 41 wild grass carp parents

Locus	Number of alleles (NA)	Effective number of alleles(NE)	Observed heterozygosity (HO)	Expected hetetrozygosity (HE)	Polymorphism information content (PIC)
G023	14	5.4422	0.8500	0.8266	0.7955
G5002	12	6.2841	0.8293	0.8512	0.823
G5004	10	6.7375	0.9024	0.8621	0.8338
G5006	7	5.5940	0.8049	0.8314	0.7974
G5010	12	5.9716	0.8293	0.8428	0.8124
G5011	14	7.6063	0.9512	0.8793	0.856
G5012	11	8.2604	0.8537	0.8898	0.8667
G5020	11	8.1124	0.8421	0.8884	0.8647
G5023	12	7.6934	0.8049	0.8808	0.8569
G5024	11	5.5296	0.9512	0.8293	0.7958
G5025	15	10.5723	0.9024	0.9166	0.8976
G5028	7	3.1391	0.7317	0.6899	0.6442
G5034	14	7.9668	0.8537	0.8853	0.8616
G5035	12	7.4053	0.8780	0.8756	0.8516
G5036	11	4.4881	0.8000	0.7870	0.7546
G5037	13	9.5783	0.9268	0.9067	0.8865
Average	11.625	6.8988	0.857	0.8527	0.8249

### Components of the random mating population

**Figure 1:**
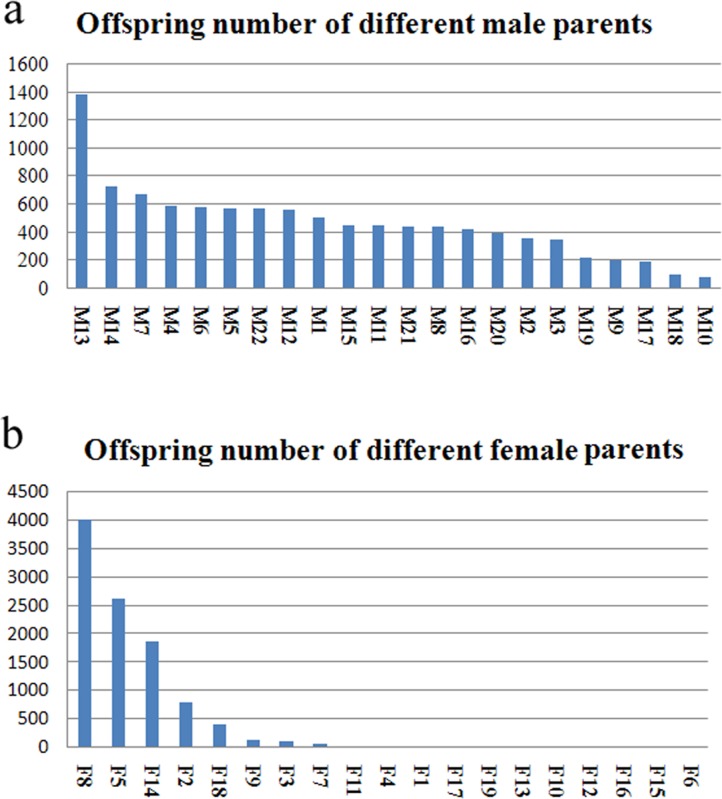
Number distribution of the half-sib families of male and female parents **a.** the offspring number of different male parents; **b.** the offspring number of different female parents.

The weight distribution of 10,245 individuals in the growth experimental group was not a normal distribution, and the average weight was 8.05±7.24g. Paternity test of the 10,245 offspring showed that all 22 male parents obtained offspring. Most of the male parents (16, accounting for 72.73%) produced offspring ranging from 300 to 800, and individual male parent exhibited significant difference in their numbers of offspring (See Figure 1a). Female parents also showed discrepancies in their number of offspring. The female F8 produced the most offspring (4,010), accounting for 39.14% of the total offspring number. Only seven females produced more than 100 offspring, while the remaining females produced less than 100 offspring (see Figure 1b). From 10,245 offspring, 270 fish families were resolved, most of which had few offspring. Seventy-eight families resulted in 30 fish or more (FF1-FF78; 28.89%), which were produced by 22 male parents (M1-M22) and five female parents (F2, F5, F8, F14 and F18; see Table 5).

### Comparative analysis of body weight traits in different families

**Figure 2:**
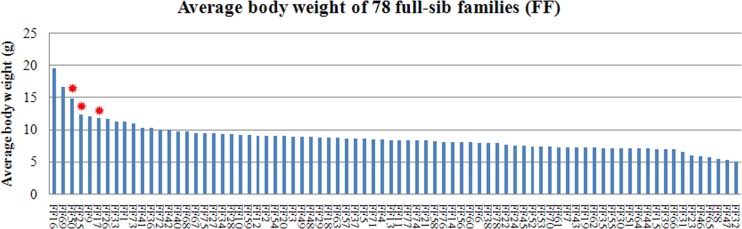
Distribution of average body weight in 78 families The red asterisks indicate three families with higher body weight.

The body weights of fish in the 78 families with 30 or more offspring were statistically analyzed. The estimate of individual heritability for body weight using the animal model was 0.11 ± 0.04; paternal heritability and maternal heritability were 0.10 ± 0.03 and 0.12 ± 0.05 respectively. The Kruskal-Wallis test and median test showed that body weights in three families FF17 (M5 × F5), FF25 (M7 × F14) and FF50 (M14 × F14) were high, with an average of 11.77 g, 12.26 g and 14.80 g, respectively (see Figure 2). Despite the average weight of FF16 and FF69 were higher, these two families did not differ statistically significantly in body weight. Analysis of the weight distribution of individuals in these two families suggested that the average weight was mainly affected by some discrete large values of individuals.

### Comparative analysis of GCRV resistance traits in different families

**Figure 3:**
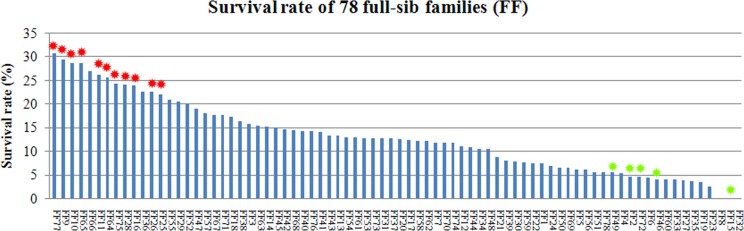
Distribution of survival rate in 78 families The red asterisks indicate 11 families which were significantly higher than the average survival rate of the total population (*P* < 0.01, df = 1). The green asterisks indicate 5 families (L2: 4.55%, L15: 0.00%, L46: 4.06%, L49: 5.39% and L72: 4.46%) were significantly lower than the average survival rate of the total group (*P* < 0.01, df = 1).

In the GCRV infection experimental group, 1,311 of the 10,000 fish survived, resulting in an average survival rate of 13.11%. Paternity test of surviving individuals (1,311 fish) showed them was from 128 families including the families FF1-FF78. Through the previous paternity test of the growth experimental population (10,245 fish), we obtained the individual numbers of fish from the families FF1-FF78 in 10,245 fish. Based on the ratio of the individual numbers of fish from families FF1-FF78 in 10,245 fish, we computed the initial individual numbers of fish from the families FF1-FF78 in the infection experimental population (10,000 fish). Thus the survival rate of the families FF1-FF78 in the infection experimental population was calculated. The estimate of heritability for survival using the animal model was 0.63 ± 0.11. The chi-square test showed that survival rates in 11 families (FF9: 29.29%, FF10: 28.61%, FF11: 26.02%, FF16: 23.75%, FF25: 22.03%, FF26: 22.46%, FF28: 23.97%, FF64: 25.60%, FF65: 28.57%, FF75: 24.25% and FF77: 30.74%) were significantly higher than the average survival rate of the total population (*P* < 0.01, df = 1). Furthermore, the survival rates in 5 families (FF2: 4.55%, FF15: 0.00%, FF46: 4.06%, FF49: 5.39% and FF72: 4.46%) were significantly lower than the average survival rate of the total group (*P* < 0.01, df = 1; see Figure 3).

## DISCUSSION

An artificial random mating population allows convenient large-scale identification and selection of families. In one experiment, many families can be identified so that the working efficiency is substantially improved, and a completely identical test environment improves the confidence and accuracy of the test results. Furthermore, the test facilities required are reduced, thereby limiting the intensity of farming management. However, random mating population may cause the subsequent experimental costs increase, such as the late DNA preparation and paternity test. In the DNA sample preparation process, we could take the Chelex-100 boiling method (one operation can extract 96 samples) to save cost. In paternity test, the costs and efficiency could be further improved through a wide screening of microsatellite loci and optimized design of multiplex PCR.

In our random mating population, the theoretic number of families was 418 (19 × 22). Of these, 270 families with 10,245 offspring were actually tested. Due to a low number of offspring in most families of the experimental population, only 78 valid families (with 30 or more offspring) could be statistically analyzed. Different fertilization rates of the female eggs and early survival rates of juveniles in different families might be the major causes of the heterogeneous distribution of the numbers of offspring in different families. The test results (Figure 1) showed that most male parents had similar numbers of offspring number, with the exception of one individual (M13). On the other hand, the female parents differed significantly in their number of offspring; only seven females produced more than 100 offspring. Previous studies have shown that egg quality has an impact on the fertilization capacity and survival of juveniles, especially in the transitional stage, when the juvenile fish change from endogenous to exogenous nutrition, in what is also known as the “sensitive period” or “dangerous period” [25]. This phenomenon has been reported in *Nibea miichthioides* [26], *Pseudosciaena crocea* [27], and *Paralichthys olivaceus* [28]. When designing a random mating population, these factors should be comprehensively taken into account. Thus, it may be more appropriate to mix juveniles surviving the “dangerous period” (instead of fertilized eggs) in equal amounts for construct the experimental population. This would reduce the discrepancy in the offspring numbers in the experimental population and improve the efficiency of further tests.

Heritability of breeding populations of target characters is an important parameter for evaluating sustainability of breeding. There is little literature regarding the genetic parameters for body weight and disease resistance in grass carp. This study found that body weight traits in grass carp showed low heritability (h^2^<0.2), and disease resistance traits in grass carp showed high heritability (h^2^>0.3). Heritability of growth performance traits in grass carp (0.11 ± 0.04) was similar to that in *Macrobrachium rosenbergii* (0.11 ± 0.08) [29], but lower than the heritability of growth trait in *Oreochromis niloticus* (0.31) and in *Oreochromis shiranus* (0.31-0.35) [30]. Grass carp with low growth heritability may be caused by that the weight distribution of grass carp random mating population was too deviated from normal distribution. Heritability of GCRV resistance traits in grass carp (0.63 ± 0.11) was higher than values (0.12-0.18) reported in the bacterial resistance study in farmed fish (*Onchorhynchus mykiss*, *Salmo salar*, *Labeo rohita*, *Gadus morhua*, *Sparus aurata*), and was similar to values reported (0.4-0.79) in the viral resistance study in farmed fish (*Onchorhynchus mykiss*, *Salmo salar*, *Gadus morhua*, *Cyprinus carpio*) [31-39]. Some researchers have proposed that mode of infection, infectious agent, level of mortality, and implemented statistical models are extremely variable across different studies, hindering a proper comparative analysis of obtained results. The latter more closely mimic natural infection may be more conducive to our comparative study of heritability of disease resistant among different fish [39].

The statistical analysis of body weight and disease resistance traits in 78 families showed that growth and GCRV resistance traits were different in wild grass carp families. This showed that abundant genetic diversity exists in wild grass carp germplasm resources, and that the breeding of improved grass carp varieties has a good genetic basis. Our results showed that the family FF25 inherited from its parents M7 and F14 both the traits for fast growth and GCRV resistance. Thus, base populations for breeding generated from M7 and F14 could be used for improving both growth and disease resistance traits. In addition, we found that six families (FF9, FF10, FF11, and FF25, FF26, FF28) of the GCRV-infected experimental population had a significantly higher survival rate. These six families were half-sib families derived from the parental males M3 and M7, respectively (see Table 5). This suggests the existence of a “dominant” disease-resistant gene in the genomes of M3 and M7, which offers the possibility of exploiting their resistance-based gene/marker through QTL or GWAS analysis using the populations generated from M3 and/or M7.

In conclusion, our results confirmed that the germplasm resources of wild grass carp have high genetic diversity. Breeding of GCRV resistant strains of grass carp have better genetic basis. The results of this study provide a basis for constructing basal populations for grass carp selective breeding and QTL and GWAS analysis.

**Table 5:** Parental composition of 78 full-sib families and their individual numbers in growth experimental population (10,245 fish)

Full-sib families (FF)	F2	F5	F8	F14	F18
M1	FF1(42)	FF2(135)	FF3(195)	FF4(98)	-
M2	FF8(30)	FF5(85)	FF7(131)	FF6(71)	-
M3	-	FF9(70)	FF11(126)	FF10(86)	-
M4	FF15(50)	FF13(169)	FF14(202)	FF12(113)	-
M5	FF19(30)	FF17(125)	FF18(261)	FF16(82)	-
M6	FF23(41)	FF21(166)	FF22(209)	FF20(98)	FF24(30)
M7	FF27(53)	FF26(146)	FF28(312)	FF25(107)	-
M8	FF32(35)	FF30(118)	FF31(186)	FF29(65)	-
M9	-	FF34(59)	FF35(58)	FF33(52)	-
M11	FF39(39)	FF37(130)	FF38(171)	FF36(77)	-
M12	-	FF41(153)	FF40(246)	FF42(91)	-
M13	FF46(101)	FF44(379)	FF43(507)	FF45(273)	FF47(49)
M14	FF51(56)	FF49(171)	FF48(306)	FF50(126)	FF52(36)
M15	FF56(34)	FF54(111)	FF53(209)	FF55(59)	-
M16	-	FF58(93)	FF57(183)	FF59(82)	-
M17	-	FF60(77)	FF61(40)	FF62(34)	-
M18	-	-	FF63(47)	-	-
M19	-	FF64(68)	FF65(61)	FF66(38)	-
M20	FF70(35)	FF69(79)	FF67(163)	FF68(86)	-
M21	FF74(35)	FF72(115)	FF71(163)	FF73(97)	-
M22	FF78(56)	FF76(138)	FF75(224)	FF77(100)	-

## MATERIALS AND METHODS

### Construction of random mating population

The experimental population was constructed by randomly mating 19 female (F1-F19) and 22 male (M1-M22) grass carp parents from different water systems at the end of April (see Table 1) in the Guanqiao Experimental Station of the Institute of Hydrobiology, Chinese Academy of Sciences (Wuhan, China). The same amount (about 5 mL) of mixed semen fertilized the same amount (about 2 L) of eggs from each female. The fertilized eggs were hatched in the same hatchery pond for emerging fry. Ethical approval for the work and Field permits for the collection of fish were obtained from Expert Committee of Biomedical Ethics, Institute of Hydrobiology of the Chinese Academy of Sciences. The Reference number obtained was Y11201-1-301.

### Growth experiment

Over 20,000 fry were selected randomly and bred in a pond of an approximately 2,600 m^2^ located in the Guanqiao Experimental Station of the Institute of Hydrobiology, Chinese Academy of Sciences (Wuhan, China). At the end of July, 10,245 fish aged 3 months were weighed and placed in 10 liters of water (containing 0.1ml clove oil per liter of water) for anesthetic. 10 minutes later, about 0.5 cm^2^ caudal fin of each fish was quickly removed using a pair of scissors and cryopreserved in 96-well plates at −20°C for DNA sample preparation.

### Viral infection experiment

The fish with obvious symptoms (hyperaemia in muscle, operculum and fin) after infection with GCRV were collected, and then to be euthanized in water containing a high dose of clove oil (0.5 ml clove oil per liter of water). The fishes whose gills had not moved for 10 minutes or even longer were considered to be dead. The intestines and bones of dead fish were removed. After weighing, a three-fold volume of 0.7% saline solution was added and the tissues were ground, incubated at 28°C for 2 h and filtered with gauze. The filtrate was mixed evenly with the floating feed in 1:1 (g/mL) for viral infection feeding.

A total of 10,000 fry were randomly selected and bred in another pond of about 1,300 m^2^. Here, viral infection was performed at an age of 5 months (at the end of September) by feeding two times per day. This period lasted 3 days at a water temperature of 25-28°C. After an incubation period of 6 days, the fish showed symptoms of infection, and the duration of the disease lasted 4 weeks. During this period, the health of the fry was checked every 4 hours. The belly-up fry which floated in the water with slowly breathes were removed out to be euthanized. The presence of viral RNA was detected in RNA samples from gill and intestine tissues (not shown). After continuous observation for 2 weeks, no more fish died. All remaining fish were regarded as surviving fish. The caudal fins of the surviving fish anesthetized with a low dose of clove oil as above were collected for DNA extraction.

### DNA sample preparation

The caudal fin tissue DNA samples were prepared using the one-step Chelex-100 boiling method in 96-well plates. Briefly, 150 μL 5% Chelex-100 was added to 96-well plates containing the caudal fin samples, which were then digested at 58°C for 1 h, boiled at 100°C for 8 minutes, centrifuged at 4000 rpm for 5 minutes, and 2 μL of the supernatants was collected as PCR templates for a microsatellite marker scan.

### Screening of polymorphic microsatellite loci

42 microsatellite sequences were screened from the full genome sequence of grass carp [1] with five bases in repeat unit length and a tenfold number of repeats within the arrays. With 41 parents as the experimental population, the above-mentioned 42 microsatellite loci were scanned using PCR amplification. PCR product was detected and typed using LI-COR 4300 DNA automatic gel electrophoresis system (LI-COR Biosciences). The results indicated that there were 16 sites shown clear bands, primers of these sites were shown in Table 2 (GenBank accession numbers: KJ930016-KJ930031).

### Multiplex PCR detection

Multiplex PCR detection method was used for the microsatellite loci genotyping of offspring individuals. Eight microsatellite loci with strong amplification bands and higher polymorphism were selected. The offspring were genotyped with these eight microsatellite loci divided into three groups (3 + 3 + 2) using a multiplex PCR method (see Table 3). The PCR products were genotyped by electrophoresis on a 3730 sequencing analyzer (Applied Biosystems). GeneScan^TM^-500LIZ○RR size standard (Applied Biosystems) was used to calculate the allele sizes. The genotyping data were analyzed using Genemapper 4.0 software (Applied Biosystems). Based on the genotyping data of the loci, genetic relationship analysis was carried out using Cervus 3 software [40] with the following parameter setup: 6 minimum typed loci and 95% confidence level.

### Statistical analysis

Genetic diversity analysis of grass carp parents: POPGEN software (Version1.32) [41] was used to analyze the number of alleles (NA), effective number of alleles (NE), observed heterozygosity (HO), expected heterozygosity (HE), and polymorphism information content (PIC) of each locus.

Paternity test: Based on all individual genotyping data, the Cervus 3 software was used to calculated the allele frequency (P), probability of exclusion (PE), the cumulative probability of exclusion (CPE), LOD value (the natural logarithm of likelihood ratio), Delta value of each locus [42]. Then we counted the likelihood ratio of possible parent of offspring, calculated the Delta value of the putative parental through the simulation program, finally guarantee the confidence level of statistical results [43].

Heritability analysis of body weight and disease resistance traits in random mating population: The models implemented in ASReml to estimate heritability for body weight and survival were two simple animal models. For body weight, the animal model is as follows:

Yijk=mu+Ti+Sj+Dk+FAMjk+Eijk

Where Yijk is the fish in ith Tank (Ti) from the cross of jth Sire cross and kth Dam and from their family FAMjk, FAMjk represents the dominant effect. Eijk is the error term.

For survival, the animal model is as follows:

Yjk=mu+FAMjk+Eijk

Where Yjk is the phenotype of the fish, FAMjk is the additive genetic effect and Eijk its environment effect.

Statistical analysis of family traits: individual body weight in the growth experimental group was analyzed using basic descriptive statistics analysis in SPSS 19 software (IBM). A comparative analysis of the body weight traits in all families was performed using non-parametric tests, including a Kruskal-Wallis test and a median test in SPSS 19 software. The significance of disease resistance traits was analyzed using a chi-square test in SPSS 19 software.
